# Oxidative stress in the pathogenesis of systemic scleroderma: An overview

**DOI:** 10.1111/jcmm.13630

**Published:** 2018-04-17

**Authors:** Rosa Vona, Antonello Giovannetti, Lucrezia Gambardella, Walter Malorni, Donatella Pietraforte, Elisabetta Straface

**Affiliations:** ^1^ Center for Gender‐Specific Medicine Biomarkers Unit Rome Italy; ^2^ Department of Clinical Medicine La Sapienza University Rome Italy; ^3^ Core Facilities Istituto Superiore di Sanità Rome Italy

**Keywords:** antioxidants, biomarkers, gender differences, oxidative stress, Raynaud's phenomenon, reactive oxidizing species, scleroderma

## Abstract

Systemic sclerosis (SSc) is a rare disorder of the connective tissue characterized by fibrosis of the skin, skeletal muscles and visceral organs. Additional manifestations include activation of the immune system and vascular injury. SSc causes disability and death as the result of end‐stage organ failure. Two clinical subsets of the SSc are accepted: limited cutaneous SSc (lc‐SSc) and diffuse cutaneous SSc (dc‐SSc). At present, the aetiology and pathogenesis of SSc remain obscure, and consequently, disease outcome is unpredictable. Numerous studies suggest that reactive oxidizing species (ROS) play an important role in the pathogenesis of scleroderma. Over the years, several reports have supported this hypothesis for both lc‐SSc and dc‐SSc, although the specific role of oxidative stress in the pathogenesis of vascular injury and fibrosis remains to be clarified. The aim of the present review was to report and comment the recent findings regarding the involvement and role of oxidative stress in SSc pathogenesis. Biomarkers proving the link between ROS and the main pathological features of SSc have been summarized.

## 1 INTRODUCTION

Scleroderma (systemic sclerosis, SSc) is an autoimmune disease characterized by vascular abnormalities that include Raynaud's phenomenon (RP), pulmonary arterial hypertension, fibrosis of skin and visceral organs. Raynaud's phenomenon, skin discoloration due to the abnormal spasm of the blood vessels causing a diminished blood supply to the local tissues,^1^ is almost always present and may be so for several years before the onset of fibrosis. Pulmonary hypertension is frequent, and anti‐centromere antibodies occur in 50%‐90% of patients.^2^

Two clinical subsets of the SSc are accepted: limited cutaneous SSc (lc‐SSc) and diffuse cutaneous SSc (dc‐SSc). The main difference between these two subsets is the speed of disease progression and the extent and severity of skin and visceral involvement in lc‐SSc; fibrosis is mainly restricted to the hands, arms and face, while dc‐SSc cases report a higher frequency of heart, lung and kidney involvement.

The disease mostly affects adult females, with a female:male ratio ranging from 3:1 to 14:1, suggesting that female sex hormones may play a role in disease pathogenesis.^3^ In the EUSTAR register, the female/male ratio was 6. Modulatory effects of oestrogens, in particular on extracellular matrix synthesis and the expression of adhesion molecules, have been observed in the fibrotic progression of the SSc process via oestrogen receptor interactions.^4^ The role of hormones in disease manifestation is also evident when considering SSc‐associated pulmonary arterial hypertension.^5^ Moreover, autoantibodies to oestrogen receptors have been found to correlate with disease activity in scleroderma patients.^6^ Although pulmonary arterial hypertension occurs more frequently in females, males with this complication show an increased frequency of other serious SSc disease manifestations, such as scleroderma renal crisis, diffuse cutaneous disease, interstitial lung disease and an increased risk of mortality.^5^

Genetic and environmental factors have been suggested to be involved in the aetiology of this disease, but the exact mechanisms involved in SSc pathogenesis are not well understood.

Several years ago Murrel 7 linked, for the first time, the pathogenesis of SSc to the occurring of oxidative stress. Several reports 8, 9 have supported this hypothesis for both lc‐SSc and dc‐SSc, although the specific role of oxidative stress in the pathogenesis of vascular injury and fibrosis remains to be clarified. Reactive oxygen and nitrogen species (here collectively called reactive oxidizing species, ROS) are considered the background pathology involved in the development of this disease, and heavily contribute to the clinical manifestations associated with SSc. Indeed, ROS, including superoxide anion (O_2_^•^), hydrogen peroxide (H_2_O_2_), hydroxyl radicals (^•^OH), nitric oxide (^•^NO), peroxynitrite (ONOO^−^) and hypochlorous acid (HOCl), can stimulate the production of pro‐inflammatory and pro‐fibrotic cytokines (such as PDGF and TGF‐β), induce proliferation and activation of fibroblasts, increase the synthesis of type I collagen and promote vascular dysfunction.^8^, ^9^ Studies on animal models have further strengthened the hypothesis of a role of oxidative stress in the onset and course of this disease. Skin changes typical for lc‐SSc and dc‐SSc have been induced in vivo by the intradermal injection of ROS‐generating compounds.^10^, ^11^, ^12^ In addition, targeting of ROS‐generating NADPH oxidase in vitro and in vivo suppresses fibroblast activation and experimental skin fibrosis.^13^

## 2 EVIDENCE OF OXIDATIVE STRESS IN SSC PATIENTS

Experimental evidences suggested the occurrence of oxidative stress in vivo in SSc patients. Samples of either fibrotic or apparently normal skin from patients with dc‐SSc showed higher ROS levels, in particular O_2_^•^, as compared to healthy control skin, suggesting that ROS could be initial or intermediate in terms of disease time‐line progression.^14^ High ROS levels, mainly O_2_^•^ and ^•^NO, have been measured ex vivo in cells from SSc patients, for example in fibroblasts,^15^, ^16^ monocytes,^17^ T lymphocytes^18^ and erythrocytes.^19^ Likewise, the total oxidant status of serum from SSc patients has been found to be higher with respect to control samples.^20^

Quite high oxidative stress biomarkers in circulating blood have been found in SSc patients. In a recent meta‐analysis involving 47 studies published up to the end of 2015, Luo et al^21^ reported increased levels of malondialdehyde (MDA), lipid hydroperoxides and endogenous ^•^NO inhibitor asymmetric dimethylarginine (ADMA) in plasma of patients with SSc with respect to healthy controls. MDA is a stable marker specific to omega‐3 and omega‐6 fatty acids peroxidation. In a case–control study, an inverse relationship was found between high plasmatic MDA levels and disease duration.^22^ Riccieri et al^23^ found that the increased hydroperoxide levels measured in plasma of SSc patients correlated with the capillaroscopy semiquantitative rating scale score (predictive of novel future severe organ involvement in SSc), and with the rating system for avascular areas. These authors also reported that the levels of carbonyl groups, biomarkers of protein oxidation, inversely correlated with modified Rodnan's skin score, a standard outcome measure for skin disease in SSc, and were lower in patients with pulmonary fibrosis.

Increased levels of lipid hydroperoxides in cell membranes alter membrane fluidity and induce loss of membrane protein functions, and efflux of cytosolic components.^24^ The plasmatic concentration of ADMA, an endogenous inhibitor of ^•^NO synthesis, was significantly increased in dc‐SSc patients but not in lc‐SSc patients or in RP. ADMA is a novel biomarker of endothelial cell dysfunction and is recognized as an important parameter in determining cardiovascular mortality and morbidity.^25^

Another stable marker of oxidative stress is 8‐isoprostane, an eicosanoid prostaglandin‐like compound produced by the non‐enzymatic random oxidation of tissue phospholipids by ROS. 8‐Isoprostane has a potent vasoconstrictor and platelet pro‐aggregating function and stimulates endothelial cells to bind monocytes, which may promote vascular obliteration, inflammation and spasm.^26^ Elevated serum 8‐isoprostane levels in dc‐SSc and lc‐SSC were found by Ogawa et al^27^ These Authors demonstrated that the increased 8‐isoprostane levels correlated with the severity of pulmonary fibrosis, the extent of renal vascular damage and immunological abnormalities in SSc, suggesting that enhanced oxidative stress is related to the development of SSc. It has also been reported that serum levels of 8‐isoprostane in SSc patients positively correlate with serum heat shock protein 70 (Hsp70) levels.^28^ Increased levels of Hsp70 in SSc patients have been associated with pulmonary fibrosis, skin sclerosis, renal vascular damage, oxidative stress and inflammation.^28^

Oxidative post‐transcriptional protein modifications, for example, nitrated proteins, known to be a marker of ^•^NO‐derived oxidants,^29^ and advanced oxidation protein products (AOPP) have been measured in SSc plasma and skin.^30^ Interestingly, AOPP in turn seems to be able to stimulate ROS formation in cell targets. Indeed, serum containing high AOPP levels isolated from SSc patients has been found to stimulate endothelial cells and fibroblasts from healthy donors to generate ROS involved in vascular or fibrotic complications.^30^ Interestingly, AOPP generated by different oxidation patterns can induce the selective triggering of cells to produce H_2_O_2_ or ^•^NO, suggesting that these oxidation products may be involved in the generation of different types of ROS in SSc patients.^30^ Moreover, urinary levels of both 8‐hydroxy‐2′deoxyguanosine (the main validated biomarker of endogenous oxidative damage to DNA) and F2‐isoprostane (a product of ROS‐mediated arachidonic acid peroxidation) were found to be higher in SSc patients than in controls.^31^ The multivariate analysis indicated a relevant association with a fibrotic phenotype. In fact, 8‐hydroxy‐2′deoxyguanosine levels were significantly associated with the presence of pulmonary fibrosis, decreased forced vital capacity and decreased alveolar volume suggesting a potential predictive value of this biomarker.^31^ Biomarkers of oxidative stress detected in SSc are listed in Table 1.

**Table 1:** Biomarkers of oxidative stress in scleroderma (SSc)

Biomarkers	Features	Specificity in SSc
Malondialdehyde (MDA)	Marker specific of omega‐3 and omega‐6 fatty acids peroxidation	High plasmatic MDA levels inversely correlate with disease duration
Asymmetric dimethylarginine (ADMA)	Endogenous inhibitor of ^•^NO synthesis, marker of endothelial cell dysfunction	Significantly increases in dc‐SSc patients but not in lc‐SSc patients or in RP
8‐isoprostane	Produced by the non‐enzymatic random oxidation of tissue phospholipids by oxygen‐derived radicals	Correlates with the severity of pulmonary fibrosis
Advanced oxidation protein products (AOPP)	Generated by different oxidation patterns, stimulate ROS production	Favour vascular or fibrotic complications
8‐hydroxy2deoxyguanosine	Marker of endogenous oxidative damage to DNA	Associated with the presence of pulmonary fibrosis, decreased forced vital capacity and decreased alveolar volume
F2‐isoprostane	Product of free radical‐mediated arachidonic acid peroxidation	Associated with a fibrotic phenotype
Nitrated proteins	Product of ^•^NO‐mediated protein oxidation	Associated with the severity and duration of the disease

## 3 PLASMA ANTIOXIDANT CAPACITY IN SSC

The total antioxidant capacity (TAC), which accounts for the ability of tissues to counteract oxidative stress, has been reported to be decreased in plasma of SSc patients.^14^, ^15^, ^23^, ^32^ The plasma components responsible for the antioxidant status are albumin, uric acid, ascorbic acid, α‐tocopherol, β‐carotene, bilirubin, glutathione, cysteine, dihydrolipoate and ubiquinol. TAC is modulated by the free radical load, metal concentration and dietary intake of antioxidants and results from the balance between oxidant species and plasma antioxidants. The low level of TAC could be the consequence of an increased ROS production determined by chronic inflammation or by other pathological conditions such as ischaemia,^32^ or it could be due to multiple gastrointestinal manifestations (dysmotility, decreased pancreatic function, bacterial overgrowth and malabsorption of fat) that lead to the reduction in nutrient uptake and the increase in nutrient losses. In this regard, Herrick et al measured decreased levels of selenium and ascorbic acid independent on dietary regimen, with lc‐SSc patients having even lower levels than dc‐SSc patients.^33^

Interestingly, a possible role of autoantibodies in the reduction of tissue antioxidant capacity has been hypothesized after the finding of antibodies directed towards ROS detoxifying enzymes, for example, superoxide dismutase,^34^ peroxiredoxin^35^ and methionine sulphoxide reductase^36^ in the plasma of SSc patients. Moreover, in an animal model of bleomycin‐induced SSc, subcutaneously injected, bone marrow‐derived, mesenchymal stem cells expressing thioredoxin 1 attenuated skin fibrosis and oxidative stress.^37^

## 4 ROS SOURCES IN SSC

**Figure 1:**
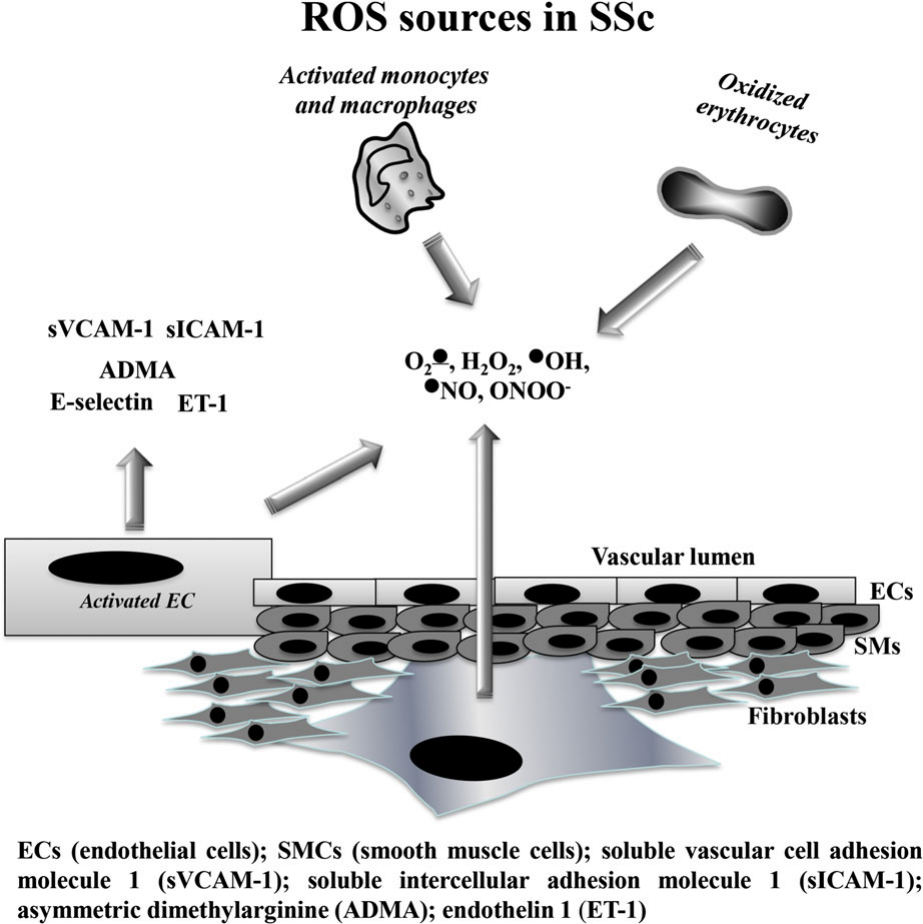
Sources of reactive oxidizing species (ROS) in SSc. In SSc, ROS may be generated inside the vascular lumen by activated monocytes and macrophages, erythrocytes, endothelial cells and fibroblasts. In addition to ROS, activated endothelial cells release adhesion molecules, which can trigger an inflammatory cascade, elevating the plasma levels of pro‐inflammatory cytokines in patients

Ischaemia reperfusion events, clinically manifested in SSc patients as RP, have been considered the most likely contributors to the abnormal oxidative stress observed in this disorder.^8^ ROS production in SSc tissues could also be triggered by the interaction of cytokines or growth factors with their specific receptors, such as interleukin‐6 (IL‐6), IL‐3, tumour necrosis factor α, angiotensin II, platelet‐derived growth factor (PDGF), transforming growth factor β1 (TGF‐β1), nerve growth factor, fibroblast growth factor, and granulocyte–macrophage colony‐stimulating factor.^3^, ^8^ ROS may be generated inside the vascular lumen by peripheral blood cells, or within the vessel wall by monocytes, endothelial cells, erythrocytes and adventitial fibroblasts in response to one or more noxious agents (Figure 1).^38^ Cells involved in ROS production are reported below.

### 4.1 Monocytes

Sambo et al demonstrated that monocytes from SSc patients spontaneously release higher levels of O_2_^•^ than monocytes from patients with primary RP or from healthy donors. The generation of O_2_^•^ is sustained through the activation of NADPH oxidase that can be triggered by cytokines such as IL‐1, IL‐6, IL‐8 and TNF‐α, whose levels have been found to increase in SSc patients.^17^

### 4.2 Endothelial cells

Vascular injury and organ devascularization are among the major features of SSc. However, the factors that induce endothelial dysfunction in SSc are still unclear. There are several serological biomarkers that reflect the vasculopathy of the disease: ROS, endothelin 1 (ET‐1), cell adhesion molecules (eg, E‐selectin) and anti‐endothelial antibodies. ^•^NO, the potent vasodilator synthesized from L‐arginine by ^•^NO synthase (NOS), is considered to have a biphasic effect in physiological and pathological conditions, being both beneficial and detrimental depending on the concentration and local environment. Kundu et al^39^ measured the bioactive forms of ^•^NO (nitrothiols) in SSc and found that their levels were very low in plasma of patients with RP and SSc. With respect to dc‐SSc patients, plasma of patients with primary RP or lc‐SSc contained higher concentrations of nitrite and nitrate, two end‐products of ^•^NO metabolism.^29^ On the other hand, plasma of patients with dc‐SSc contained higher levels of both ADMA and nitrated proteins, an irreversible post‐transcriptional protein modification induced by the ^•^NO‐derived nitrating agents (peroxynitrite, nitrogen dioxide radical). Interestingly, the high levels of nitrated proteins were strongly associated with the severity and duration of dc‐SSc disease.^29^ Thus, abnormal ^•^NO metabolism in the plasma of RP, lc‐SSc and dc‐SSc patients may reflect the underlying endothelial damage and vasculopathy of SSc diseases. ET‐1 is produced by endothelial cells and is a key mediator of vasculopathy. It promotes cell growth, arterial wall thickening and endothelial cell dysfunction, resulting in decreased levels of nitric oxide. Increased ET‐1 production can trigger an inflammatory cascade, raising the plasma levels of pro‐inflammatory cytokines in patients with pulmonary arterial hypertension.^40^ ET‐1 is over‐expressed in both early‐ and late‐stage SSc, as shown by the elevated levels found in the blood vessels, lungs, kidneys and skin of SSc patients.^41^ Adhesion molecules as E‐selectin, sICAM‐1 (intercellular adhesion molecule 1) and sVCAM‐1 (vascular cell adhesion molecule 1) are significantly elevated in SSc patients. E‐selectin is produced by the activated endothelium. Its soluble forms predominate in patients with dc‐SSc vs those with lc‐SSc.^42^ sVCAM‐1 is primarily released from endothelial cells, but can be produced by epithelium, dendritic cells and macrophages. Soluble ICAM‐1 can be produced by a variety of cells including epithelium, leucocytes, hepatocytes and smooth muscle cells. In SSc, VCAM‐1 has been found expressed in endothelial cells and in skin biopsy specimens,^43^ while ICAM‐1 expressed in fibroblasts,^43^, ^44^ and infiltrating mononuclear cells.^45^ Anti‐endothelial antibodies (AECAs) are a heterogeneous class of antibodies that have been identified in the serum of 44%‐84% of SSc patients.^46^ AECAs are able to activate endothelial cells (EC), induce the expression of adhesion molecules and trigger EC apoptosis in the presence of NK cells in vitro.^47^

### 4.3 Red blood cells

Because of their high iron concentration (~20 mmol/L), red blood cells (RBCs) can be considered as an “iron mine” but, paradoxically, they also represent one of the major components of blood antioxidant capacity and one of the cells with higher resistance to oxidative stress.^48^ Crossing inflamed areas, RBCs can help to detoxify ROS, thus rescuing or partially “protecting” cells (eg, endothelial cells). A completely different situation may arise when RBCs cross a tissue where an intense production of ROS occurs. Under these conditions, RBCs may accumulate oxidative damage, and act as pro‐oxidant “bullets” capable of modifying the behaviour and fate of endothelial cells.^49^ It has been demonstrated that the systemic oxidative imbalance occurring in SSc patients induces changes in RBCs, including cytoskeleton oxidative denaturation and derangement, and loss of lipid asymmetry.^6^ These changes can result in the alteration of RBC adhesive properties, aggregability and deformability, and correlate with disease severity. In agreement, high MDA and ^•^NO levels have been measured in RBCs of SSc patients.^19^

### 4.4 Fibroblasts

Apart from maintaining the mechanical properties of the skin, dermal fibroblasts take on an important role in the process of wound healing by synthesizing collagen and relevant cytokines such as keratinocyte growth factor. Fibroblasts from patients with SSc constitutively produce a greater amount of ROS such as O_2_^•^ and H_2_O_2,_ which are responsible for the increased synthesis of type I collagen. It has been demonstrated that cultured SSc fibroblasts show an abnormal phenotype that is characterized by the overproduction of different extracellular matrix (ECM) proteins and display increased constitutive expression of pro‐inflammatory factors, for example, IL‐6 and IL‐1, and soluble factors that mediate trans‐endothelial migration of mononuclear cells.^50^ In spite of the high production of ROS, scleroderma fibroblasts are resistant to Fas‐induced apoptosis. Probably because they have increased levels of Bcl‐2 and decreased levels of Bax. Bcl‐2 is an anti‐apoptotic, and Bax is a pro‐apoptotic member of the Bcl‐2 family.^51^ Moreover, it has been demonstrated that both cellular Abelson (c‐Abl) and TGF‐β1 serve as apoptosis suppressors in human dermal fibroblasts. c‐Abl is known as a TGF‐β1‐modulating molecule in fibrosis. TGF‐β1 is an important cytokine that induces fibroblast differentiation into myofibroblasts and is considered to be the major factor towards chronic fibrosis. SSc patients express elevated TGF‐β1 levels in the early lesions, but not in established fibrotic tissue.^52^ Kim and collaborators demonstrated that TGF‐β1 induced the activation of Akt in normal and RA synovial fibroblasts, and that TGF‐β1 exerted its anti‐apoptotic effect, in part, through the PI3 kinase/Akt pathway.^53^

## 5 ANTIOXIDANT THERAPY FOR SSC

A reduced concentration of classical antioxidants, such as antioxidant vitamins (ascorbic acid, α‐tocopherol, and β‐carotene) and minerals (zinc, selenium), has been found in RP and SSc. This antioxidant potential deficiency increases the propensity to oxidative stress, favouring the development of injury mediated by ROS generation. Currently, molecules as N‐acetyl‐l‐cysteine (NAC), antioxidant vitamins and polyphenols may be useful in the supportive therapy of SSc and RP. NAC acts as a precursor for the substrate (l‐cysteine) in the synthesis of hepatic glutathione (GSH), and replenishes GSH in deficient cells. It influences protein thiols, supports glutathione synthesis and generates free sulfhydryl groups. In many studies, NAC has shown a beneficial influence on SSc, diminishing cellular ROS in fibroblast and replenishing free cellular thiols.^54^ Vitamin E is a potent intracellular antioxidant^55^ and protects the polyunsaturated fatty acids present in membrane phospholipids and in plasma lipoproteins. Moreover, its administration considerably increases cell‐mediated and humoural immune functions in human.^56^ Polyphenols are natural antioxidants present in many plant foods. Among these, (−)epigallocatechin‐3‐gallate (EGCG) present in green tea extracts (*Camellia sinensis*) is effective in eliminating oxidative stress in SSc. It is a scavenger of free radicals, inhibits the formation of ROS such as O_2_^•^, and peroxynitrite and reduces oxidative stress.^57^ However, some clinical trials have shown that the treatment with antioxidants has had limited success. Literature data report that: (i) a 3‐week vitamin E treatment at doses of 500 or 1000 mg/day neither reduced the basal rate of lipid peroxidation nor improved microvascular perfusion following cold exposure^56^; (ii) ascorbic acid did not improve endothelial vasomotor dysfunction in the brachial circulation of patients with RP secondary to systemic sclerosis^58^; and (iii) no clinical benefit was observed in SSc patients after an active treatment with a combination of micronutrient antioxidants (selenium, beta‐carotene, vitamin C, vitamin E and methionine) and allopurinol.^59^

There are several possible explanations for these poor results, for example, (i) the short duration of therapy. It is possible that in order to be effective, the antioxidant therapy has to be given early in the SSc disease process, before the onset of irreversible tissue damage; (ii) the malabsorption syndrome resulting from increased collagen deposition in the intestines or bacterial overgrowth in SSc patients; and (iii) an altered renal clearance of the aqueous phase antioxidants (such as ascorbic acid) and its increased excretion in SSc patients.

Although recent findings supporting the efficacy of antioxidant therapy are very encouraging,^54^, ^55^, ^56^, ^58^ further studies are necessary to define the therapeutic potential of the analysed antioxidants in SSc.

Anyway, notwithstanding substantial advancements, the morbidity and mortality in SSc are still high and can largely be attributed to a delay in diagnosis. In fact, at the time of diagnosis, SSc is often well established with significant irreversible tissue and organ damage. An early and accurate diagnosis of SSc and the use of autoantibody testing embedded in evidence‐based clinical care pathways will help improve SSc‐associated clinical outcomes and healthcare expenditures.

## 6 CONCLUSIONS

This review comments on the recent findings regarding the involvement and the role of oxidative stress in SSc pathogenesis. Biomarkers proving the link between ROS and the main pathological features of SSc could be of interest in the development of more appropriate diagnostics and therapeutic strategies. In fact, the pathogenesis of SSc appears as complex and involves a complex framework of “actors” such as endothelial cells, epithelial cells, fibroblasts and immunological mediators, resulting in dysregulated vascular remodelling and, ultimately, vasculopathy. In addition, taking into account the significant gender disparity in the occurrence of the disease as well as the well‐known differences in ROS generation by XX and XY cells, including vessel cells,^60^ the relevance of the redox state as a gender‐associated determinant in the disease onset and progression cannot be ruled out.

## CONFLICT OF INTEREST

The authors declare no commercial or financial conflict of interest.
